# The effect of pupil size on data quality in head-mounted eye trackers

**DOI:** 10.3758/s13428-025-02880-3

**Published:** 2025-12-03

**Authors:** Mohammadhossein Salari, Diederick C. Niehorster, Marcus Nyström, Roman Bednarik

**Affiliations:** 1 School of Computing, University of Eastern Finland, Joensuu, Finland; 2 Lund University Humanities Lab, Lund University, Lund, Sweden; 3 Department of Psychology, Lund University, Lund, Sweden

**Keywords:** Eye tracking, Pupil size artifact (PSA), Head-mounted eye trackers, Data quality

## Abstract

Changes in pupil size can lead to apparent gaze shifts in data recorded with video-based eye trackers in the absence of physical eye rotation. This is known as the pupil-size artifact (PSA). While the PSA is widely reported in desktop eye trackers, it is unknown whether and to what extent it occurs in head-mounted eye trackers. In this paper, we examined the effects of pupil size variations on eye-tracking data quality in four head-mounted eye trackers: the Pupil Core, the Pupil Neon, the SMI ETG 2w, and the Tobii Pro Glasses 2, in addition to a widely used desktop eye tracker, the SR Research EyeLink 1000 Plus. Participants viewed a central target on a monitor while we systematically varied the screen brightness to induce controlled pupil size changes. All head-mounted eye trackers exhibited PSA, with apparent gaze shifts ranging from 0.94$^\circ$ for the Pupil Neon to 3.46$^\circ$ for the Pupil Core. Except for the Pupil Neon, all eye trackers exhibited a significant change in accuracy due to pupil size variations. Precision measures showed device-specific effects of pupil size changes, with some eye trackers performing better in the bright condition and others in the dark condition. These findings demonstrated that, just like desktop eye trackers, head-mounted video-based eye trackers exhibited PSA.

## Introduction

Video-based eye trackers have become the most commonly used technology for monitoring eye movements (Duchowski, 2017; Larrazabal et al., 2019). These systems often use the pupil center and other eye image features such as the corneal reflection to determine gaze direction (Hansen & Ji, 2010; Holmqvist & Andersson, 2017). The use of the pupil as a primary feature creates a significant limitation since the pupil size never remains static (Hooge et al., 2024); its size constantly changes in response to factors such as illumination, cognitive load, and emotional state (Beatty, 1982; Laeng et al., 2012; Mathôt, 2018). In video-based eye trackers, pupil size fluctuations often lead to systematic errors in gaze estimation known as the pupil-size artifact (PSA). PSA is defined by Hooge et al. (2021) as the apparent gaze deviation reported by an eye tracker during pupil size changes when the eye does not actually rotate.

This relation between pupil size and measured gaze direction has been known in eye tracking as early as 1974 (Merchant et al., 1974), but it was Drewes et al. (2012) who showed that these reported gaze shifts do not represent actual eye movements and are merely measurement artifacts in video-based systems. They conducted a systematic investigation using simultaneous recording of eye movements with the video-based SR Research EyeLink 1000 and a scleral search coil system that served as a ground-truth reference, measuring the orientation of the eyeball (also known as the bulbus oculi). In their experiment, they changed the brightness of their visual display to induce pupil size change while comparing gaze data from both systems. The video-based eye tracker reported apparent gaze shifts exceeding $2^\circ$ between these conditions. In contrast, the scleral search coil system simultaneously reported steady fixation, providing evidence that the subject’s actual gaze direction remained stable. This finding provided compelling evidence that the apparent gaze shifts were indeed artifacts of the video-based eye trackers rather than actual eye movements.

Multiple studies have investigated the PSA and quantified the magnitude of pupil size-induced apparent gaze shifts across various devices and populations. Wyatt (2010) conducted a study using an ISCAN EC-101, a 60-Hz dark pupil desktop eye tracker, with seven participants. He manipulated pupil size by alternating the screen luminance between black and white, revealing that changes in pupil size produced an average apparent gaze shift of 0.81 degrees, with a maximum shift of 1.22 degrees. Ivanov and Blanche (2011) used a tower-mounted version of an EyeLink 1000 with ten participants and employed two methods to manipulate pupil size. In their first experiment, they used bright, single-frame (5-ms) flashes on a black screen to evoke a strong pupillary light reflex, producing apparent gaze shifts of up to 1.3 degrees. In a second experiment, they modulated screen luminance across seven different conditions while subjects fixated on targets at nine screen locations, revealing apparent gaze shifts of up to 3.6 degrees. Drewes et al. (2014) investigated PSA using the SR Research EyeLink 1000 eye tracker with 39 participants. By varying background brightness to achieve pupil-dilated and pupil-constricted conditions, they reported mean apparent gaze shifts of 2.6 degrees, ranging from 0.3 to 5.2 degrees. Hooge et al. (2021) explored PSA using EyeLink 1000 Plus and Tobii Pro Spectrum eye trackers. They manipulated pupil size by changing the screen’s background slowly from black to white and back in a sinusoidal pattern while participants fixated on a target. Their results showed PSA is stable over time within participants, but differs between participants. Importantly, they found different PSA patterns between devices: the EyeLink 1000 Plus exhibited asymmetrical PSA between eyes, while the Tobii Pro Spectrum showed symmetrical PSA. The magnitude of PSA was related to the angle between the viewing direction and the camera axis. Their findings suggest that differences in eye tracker geometry can substantially impact PSA characteristics.

Prior work has suggested that brightness-driven changes in pupil size may be systematically associated with changes in vergence angle (i.e., may lead to systematic eyeball rotations) (Huckauf, 2018). Subsequent studies that independently measured pupil and corneal-reflection (CR) signals showed that, although pupil-size changes shift the estimated pupil center, the CR position remains stable, indicating that the eyeball does not rotate (Hooge et al., 2019). This supports treating PSA as a measurement error and motivates a protocol to systematically quantify its magnitude, enabling researchers to account for participant- and device-specific biases that they might face when using their eye trackers (cf. Drewes et al., 2014).

While prior studies have investigated PSA in desktop eye trackers, it is largely unknown whether pupil size changes induce apparent gaze shifts in head-mounted systems. Extensive research has evaluated various aspects of head-mounted eye tracker performance (Aziz & Komogortsev, 2022; Ehinger et al., 2019; Hooge et al., 2022; Macinnes et al., 2018; Niehorster et al., 2020; Pastel et al., 2021; Stein et al., 2021; Stuart et al., 2016; Tonsen et al., 2020; Vrzakova & Bednarik, 2012), yet only one recent study has presented a preliminary investigation of how pupil size changes affect the gaze accuracy of a head-mounted eye tracker (Salari & Bednarik, 2024).

Head-mounted eye trackers differ from desktop systems in several ways that could affect PSA characteristics. They have different optical geometries with shorter camera-to-eye distances and different viewing angles. In particular, the angle between the gaze direction and the camera, often more extreme in head-mounted setups, has been shown to be an important factor in PSA magnitude (Hooge et al., 2021). Additionally, while most video-based eye trackers use pupil-corneal reflection (P-CR) methods, implementations vary: some use multiple corneal reflections, others employ 3D eye models versus 2D feature regression, and newer appearance-based systems like the Pupil Neon bypass, to the best of our knowledge, explicit pupil detection entirely. These technical differences may lead to different PSA magnitudes and characteristics across devices.

**Table 1:** Specifications of eye-trackers used in the study based on manufacturer information

Device	Type	Rate	Accuracy	Method
EyeLink 1000 Plus	Desktop	250, 500, 1000, or 2000 Hz	0.25–0.50$^{\circ }$	P-CR; 2D feature regression
Pupil Core	Head-mounted	up to 200 Hz	0.6$^{\circ }$	P-CR; 2D feature regression or 3D eye models
Pupil Neon	Head-mounted	up to 200 Hz	1.3$^{\circ }$–1.8$^{\circ }$	End-to-end deep learning; Appearance-based methods
SMI ETG 2w	Head-mounted	60 Hz or 120 Hz	0.5$^{\circ }$	P-CR; 3D eye models
Tobii Pro Glasses 2	Head-mounted	50 Hz or 100 Hz	N/A	P-CR; 3D eye models

Participants wearing head-mounted eye trackers in real-world environments often have large and frequent pupil size changes. Additionally, many researchers who employ head-mounted eye trackers still require data with high spatial accuracy despite the inherent accuracy limitations of these devices. For example, in cartography (Fairbairn & Hepburn, 2023; Kiefer et al., 2013), researchers need to accurately determine which map features participants examine; in operating theaters, accurate gaze data reveal what anatomical structure surgeons attend to (Dik et al., 2016); and in face-to-face interaction studies, distinguishing whether participants look at eyes or mouth requires exceptional accuracy and precision (Jongerius et al., 2021; Rogers et al., 2018). In these contexts, even minor apparent gaze shifts can cause fundamental misidentifications of what participants are actually looking at, rendering results unreliable (Orquin & Holmqvist, 2018; Vehlen et al., 2022). Given the important practical and methodological implications of PSA for head-mounted eye tracking, our paper addresses these research questions: Do head-mounted eye trackers exhibit PSA? Do pupil size changes also influence other data quality measures than accuracy, such as precision and data loss?

Recent advances in eye tracking technology further highlight the need for a systematic investigation of PSA in head-mounted devices. Video-based eye trackers can be categorized according to their gaze estimation approaches: 2D feature regression and 3D eye models, which are often considered traditional approaches, and appearance-based methods (Cheng et al., 2024; Hansen & Ji, 2010; Nyström et al., 2025). The PSA effect has been well documented in traditional video eye trackers using the first two approaches, particularly those employing pupil–corneal reflection (P-CR) vectors for gaze estimation (Nyström et al., 2025). These approaches explicitly detect and measure pupil features, making them inherently susceptible to pupil size variations.

However, it remains unknown whether appearance-based systems, which are increasingly adopted in newer head-mounted eye trackers, exhibit PSA effects comparable to traditional methods or whether they are more resistant to pupil-related artifacts. Appearance-based eye trackers typically estimate gaze direction using end-to-end deep learning models that map eye images directly to gaze positions, bypassing explicit feature detection (Nyström et al., 2025). The “black box” nature of these neural network-based approaches raises our third research question: Do appearance-based head-mounted eye trackers also exhibit PSA?

To examine these questions, we manipulate participants’ pupil size by changing the screen brightness while they fixate a visual target on the screen. This experimental design allows us to measure the magnitude of apparent gaze shifts that occur due to PSA, as well as to quantify the effect of pupil size on overall accuracy, precision and data loss.

## Method

### Participants

We recruited 24 participants (17 males, seven females), all of whom were students or staff at the University of Eastern Finland. Participants had a mean age of 27.3 years ($SD = 7.13$, range 19–52 years). Prior to enrollment in the study, all potential participants underwent vision screening using a Landolt C test, and only individuals who showed a visual acuity of 1.0 or better for binocular vision were selected to participate. All participants also self-reported no existing eye conditions that could affect their eye movements. Participation was voluntary, and participants received a chocolate bar as compensation.

### Materials

#### Eye trackers

Our experimental setup included four head-mounted eye trackers as detailed in Table 1: Pupil Core (Pupil Labs, 2024a), Pupil Neon (Pupil Labs, 2024b), SMI Eye Tracking Glasses 2 Wireless (SMI ETG 2w) (SensoMotoric Instruments (SMI), 2024), and Tobii Pro Glasses 2 (Tobii, 2024). We additionally used the SR Research EyeLink 1000 Plus as a desktop reference device (SR Research Ltd, 2024), as it is a well-studied eye tracker with known PSA characteristics; it also serves to validate that our protocol reliably induces PSA.

##### Device allocation

Each participant was recorded using all available eye tracking devices during their session. All devices were used throughout the study except the Tobii Pro Glasses 2, which became available later and was therefore used in only six sessions.

**Fig. 1:**
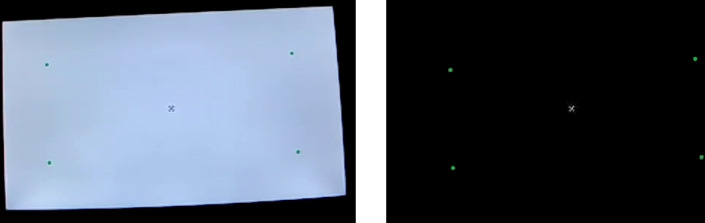
Stimulus display as captured by the Pupil Neon eye tracker: bright condition (white background, *left*) and dark condition (black background, *right*). The gray bullseye-crosshair target is visible at the center with four green reference points at the corners of the monitor

#### Stimuli and experimental design

The experiment was conducted in a windowless room with black walls to minimize visual distractions and ensure consistent ambient light conditions across all trials. Ceiling lamps remained off during the experiment, with the stimulus display serving as the only direct light source facing participants. Additional monitors necessary for experiment operation were positioned perpendicular to participants’ line of sight to minimize their influence on overall illumination reaching participants’ eyes. To ensure consistent head positioning, participants used a chin rest fixed at a distance of 930 mm from the display.

In our experiment, we manipulated pupil size through two distinct display luminance conditions: a dark condition and a bright condition. We controlled monitor luminance by presenting either a black background for the dark condition or a white background for the bright condition on the same display monitor. Ambient illuminance values were 0.11 lux for the dark condition and 21.61 lux for the bright condition, measured using a Konica-Minolta CL-500a illuminance spectrophotometer positioned at the participant’s eye level, with the CL-500a sensor facing the monitor.

On top of this background, a single target was shown at the center of the display (Samsung SyncMaster S22B350, 476 mm (w) $\times$ 268 mm (h), resolution 1920 $\times$ 1080 px). This target was a bullseye-crosshair combination with an outer diameter of $0.6^\circ$ and an inner diameter of $0.2^\circ$, following the ABC target configuration described by Thaler et al. (2013, experiment 2 in their Fig. 1). This design was used as it has been recommended by Thaler et al. (2013) for tasks requiring precise fixation. The target was rendered in gray to ensure its visibility in both experimental conditions.

To enable compensation for potential head movements during data analysis, we displayed four green circular points at the corners of the monitor throughout the experiment. These four reference points, along with the central target, are illustrated in Fig. 1.

Each recording with an eye tracker consisted of three trials. Each trial consisted of a 10-s bright period followed by a 10-s dark period. During the initial 5-s adaptation phase of both the bright and the dark periods, the target remained absent and the participants could freely look around while the pupil adapted to the luminance condition, and to allow eye trackers to automatically adjust their scene camera exposure and gain. After the adaptation period, the target was shown for another 5 s during the measurement phase, while participants were instructed to maintain steady fixation of the target.

For the EyeLink 1000 Plus, experiments were constructed using Experiment Builder version 2.5.90. For head-mounted eye trackers, we implemented a custom application using Python 3.11.8 and OpenCV 4.10.0 to display the stimuli. We ensured methodological consistency by maintaining identical timing parameters, experimental procedures, and visual stimuli across both the EyeLink and head-mounted eye tracking setups.

A Dell laptop with 13th Gen Intel Core i7-13700H 2.40 GHz CPU, 32 GB RAM, and Windows 11 Education, hereafter referred to as ‘Stimulus-PC’, was used for stimulus presentation across all eye trackers. Each eye tracker required specific hardware for data collection. The EyeLink 1000 Plus was set up in dual PC Desktop Mount configuration with the Stimulus-PC used as Display PC and a dedicated Workstation Host PC from SR Research Ltd. running Host software version 5.50. For the head-mounted eye trackers, the data collection setups were as follows: the Stimulus-PC operated the Pupil Capture software version 3.5.1 software for Pupil Core data recording. The Neon companion app version 2.8.37-prod was used for Pupil Neon data collection. For SMI ETG 2w, a ThinkPad laptop with Intel Core i7-2640M CPU 2.8 GHz and Windows 7 running iView ETG version 2.6 was utilized. Lastly, the Stimulus-PC was also used for the Tobii Pro Glasses 2 data collection using Tobii Pro Glasses Controller version 1.114.200033.

All calibrations were performed on the same stimulus display monitor used for data collection. This approach ensured consistent illumination levels and viewing distance between calibration and experimental phases. The Tobii Pro Glasses 2 and the Pupil Core require bullseye calibration targets presented on white backgrounds. Due to these requirements, we performed all device calibrations under bright conditions. While Tobii Pro Glasses 2 comes with a physical calibration card featuring precise bullseye specifications, we needed a digital solution compatible with our stimulus display approach. Therefore, we developed a custom Python script that replicated the exact Tobii bullseye target on the screen. Although the Pupil Neon and SMI ETG 2w do not require specific calibration targets, we used the same bullseye target developed for the Tobii Pro Glasses 2 for all three devices. We used the following configurations for each eye tracking device in our experiment:

- **EyeLink 1000 Plus**: 13-point calibration procedure with black calibration target circles on the white background; 500-Hz gaze sampling rate; Pupil-CR for eye tracking; Ellipse mode for pupil tracking; using the 25-mm lens; Pupil area was recorded.
- **Pupil Core**: Default calibration process using Screen Marker choreography with Marker Size 1.00 and Sampling duration parameter set to 40; 100-Hz gaze sampling rate; 3D Gaze mapping for eye tracking; Scene Camera 1280 $\times$ 720 @30 FPS.
- **Pupil Neon**: Offset correction using the Neon companion mobile application; 200-Hz gaze sampling rate; binocular eye tracking mode; Scene Camera 1600 $\times$ 1200 @30 FPS.
- **SMI ETG 2w**: One-point calibration; 30-Hz gaze sampling rate; eye tracking mode binocular; Scene Camera 1280 $\times$ 960 @24 FPS;
- **Tobii Pro Glasses 2**: Default calibration procedure but using the digitally rendered bullseye target displayed on the stimulus display instead of the physical calibration card; 50-Hz gaze sampling rate; Scene Camera 1280 $\times$ 720 @30 FPS.

### Procedure

Each participant completed testing sessions with all available eye trackers, selected in random order. For each eye tracker, we first performed calibration, or in the case of Pupil Neon, offset correction. Following calibration, participants underwent data collection consisting of three trials of the bright and dark pupil condition. Participants were given rest periods between conditions and could proceed when they felt ready. The EyeLink 1000 Plus infrared illuminator was only powered on when we were collecting data with this device and kept off for the rest of the experiment to prevent interference.

Calibration quality was verified using the following evaluation protocol. For the EyeLink 1000 Plus, the built-in validation procedure was used, and data collection proceeded if both left and right eye average errors were less than 1 degree of visual angle. For head-mounted eye trackers, participants were instructed to look at the target and four reference points while gaze accuracy was visually inspected to ensure gaze points fell within approximately 5 degrees of visual angle from the expected positions. For all eye trackers, the calibration procedure was repeated (up to three times maximum) until calibration met our quality criteria as described above. During our data collection, all participants successfully met the calibration criteria for all eye trackers within three tries.

### Data processing

For the EyeLink 1000 Plus eye tracker, recording files were exported to CSV format using DataViewer version 4.3.210. Then we replaced the invalid values with NaN in the data and converted gaze data from pixels to degrees of visual angle using Eq. (1).

1 $$\begin{aligned} \text {gaze}_{\text {degrees}} = \frac{\text {gaze}_{\text {pixels}} - \text {screen\_center}_{\text {pixels}}}{\text {resolution}_{\text {pixels/degree}}} \end{aligned}$$

where $\text {gaze}_{\text {pixels}}$ represents the recorded gaze position in pixels, $\text {screen\_center}_{\text {pixels}}$ is the pixel coordinates of the screen_center, and $\text {resolution}_{\text {pixels/degree}}$ is the conversion factor provided by the EyeLink system itself.

For head-mounted eye trackers, we first converted manufacturers’ recording file formats to a common data format using GlassesTools version 1.22.2 (Niehorster et al., 2023). After removing invalid values, we used a custom annotation tool^1^ to manually mark the start and end of each trial.

Since head-mounted eye trackers record gaze data in pixels within the scene camera’s coordinate system, we converted these measurements to degrees of visual angle relative to the screen center. This process began with detecting the target and corner reference points in the scene camera images. We implemented an image processing approach using OpenCV 4.10.0 and scikit-learn 1.5.1, which included grayscale conversion, thresholding, contour detection, and K-means clustering. Threshold values were manually selected for each eye tracker and participant to account for recording variations, with detection quality validated through visual inspection of randomly selected frames.

After detecting all screen elements, we used OpenCV in combination with the eye tracker’s camera calibration parameters to undistort both the gaze points and the screen reference points. We then calculated a scaling factor by dividing the known physical distances between reference points by their apparent distances in the undistorted scene camera image:

2 $$\begin{aligned} \text {scaling factor} = \frac{\text {physical distance}_{\text {mm}}}{\text {apparent distance}_{\text {pixels}}} \end{aligned}$$

Using this scaling factor, we converted gaze coordinates from pixels to millimeters:

3 $$\begin{aligned} \text {gaze}_{\text {mm}} = (\text {gaze}_{\text {pixels}} - \text {screen\_center}_{\text {pixels}}) \times \text {scaling factor} \end{aligned}$$

Finally, we converted these millimeter measurements to visual angles based on the viewing distance:

4 $$\begin{aligned} \text {gaze}_{\text {degrees}} = \arctan \left( \frac{\text {gaze}_{\text {mm}}}{\text {distance}_{\text {mm}}}\right) \end{aligned}$$

where $\text {distance}_{\text {mm}}$ represents the viewing distance, estimated using OpenCV and the pinhole camera model based on the known size of the four reference points and the camera’s focal length.

Further analysis was done directly on the gaze samples reported by the eye trackers. Fixation detection was not used due to differences in sampling rates and signal characteristics across the eye trackers used in this study. Applying a single fixation-detection algorithm to our data would risk introducing algorithm-specific bias and device-dependent differences in fixation-detection performance (Andersson et al., 2017; Startsev & Zemblys, 2022), which would limit between-device comparisons. We therefore applied uniform trimming and filtering procedures described below across both desktop and head-mounted eye trackers and analyzed raw gaze measurements rather than restricting analyses to fixation periods.

Visual inspection of the recorded data revealed that the middle 75% of the trials contained the period of most stable fixation behavior, with the initial and final parts of trials often containing saccades towards or away from the target. We therefore trimmed the initial and final 12.5% of each trial to exclude these inaccurate fixation episodes from the analyzed data. To address blink artifacts, we applied a two-stage filtering approach: a distance-based filter removed gaze points located more than $10^\circ$ from the target, followed by statistical outlier removal, excluding points exceeding 3 standard deviations from the mean in either dimension. This two-stage filtering process resulted in the removal of approximately 1.3% of gaze data in our study.

The final step was to convert the EyeLink 1000 Plus pupil area measure from arbitrary units to pupil diameter in millimeters. For this conversion, we used the following formula:

5 $$\begin{aligned} D_{mm} = D_{artificial} \times \sqrt{\frac{D_{recorded}}{D_{artificial\_recorded}}} \end{aligned}$$

where $D_{mm}$ represents the pupil diameter in millimeters, $D_{artificial}$ is the known diameter of an artificial pupil in millimeters, $D_{recorded}$ is the recorded pupil area in arbitrary units for participants, and $D_{artificial\_recorded}$ is the reference value from the artificial pupil recorded using the same setup^2^

### Quality metrics

All metric calculations were performed directly on raw gaze samples, without employing a fixation detection algorithm. For comprehensive descriptions of these eye tracking quality metrics, please refer to Holmqvist et al. (2012), Niehorster et al. (2020), and Nyström et al. (2025).

#### Spatial accuracy

Spatial accuracy quantifies the closeness of the recorded gaze points to the true gaze location of participants. It reflects how accurately the eye tracker can report the gaze position. A lower value of spatial accuracy indicates that the gaze points are closer to the target, suggesting better accuracy in the system.

Under the reasonable assumption that participants accurately fixated the target (Hooge et al., 2024), spatial accuracy is calculated as the mean offset between the recorded gaze point and the target location:

6 $$\begin{aligned} \text {Accuracy (degrees)} = \frac{1}{N} \sum _{i=1}^{N} d_i \end{aligned}$$

where $d_i$ is the distance between the recorded gaze point and the target location, and *N* is the total number of gaze points.

#### Apparent gaze shift

In this paper, we use the term “apparent gaze shifts” as a metric to quantify the numerical magnitude of gaze shifts recorded by the eye trackers due to PSA. The apparent gaze shift is calculated as the average Euclidean distance between the centers of gaze data collected under sequential dark and bright pupil conditions:

7 $$\begin{aligned} &  \text {Apparent gaze shift (degrees)}\nonumber \\= &  \frac{1}{n}\sum _{i=1}^{n} \sqrt{(\bar{x}{c_i} - \bar{x}{d_i})^2 + (\bar{y}{c_i} - \bar{y}{d_i})^2} \end{aligned}$$

Where *n* represents the total number of trials, $(\bar{x}{d_i}, \bar{y}{d_i})$ are the mean coordinates of the gaze points in the dark pupil condition for the *i*-th trial, and $(\bar{x}{c_i}, \bar{y}{c_i})$ are the mean coordinates of gaze points in the bright pupil condition for the *i*-th trial.

We calculated the apparent gaze shift for each pair of sequential dark and bright pupil conditions to circumvent potential gaze drift over time due to factors such as device slippage (Niehorster et al., 2020). A higher value of apparent gaze shift indicates a larger effect of PSA on the recorded gaze points.

#### Precision

Precision refers to the consistency of gaze sample measurements over time. It reflects how stable and tightly clustered the recorded gaze points are, independent of their distance from the true target location. To capture different aspects of precision, we use two complementary metrics: standard deviation (STD) and root mean square sample-to-sample error (RMS-S2S).

Standard deviation (STD) measures the overall spatial spread of the gaze samples around the centroid of gaze positions. A lower STD value indicates that the gaze points are more tightly grouped, suggesting greater precision in the reported gaze positions.

Root mean square sample-to-sample error (RMS-S2S) captures the temporal stability of the eye-tracking signal. It quantifies the average displacement between consecutive gaze samples. While STD considers the overall spread of gaze data, RMS-S2S is more sensitive to short-term fluctuations and noise in the recording.

**Fig. 2:**
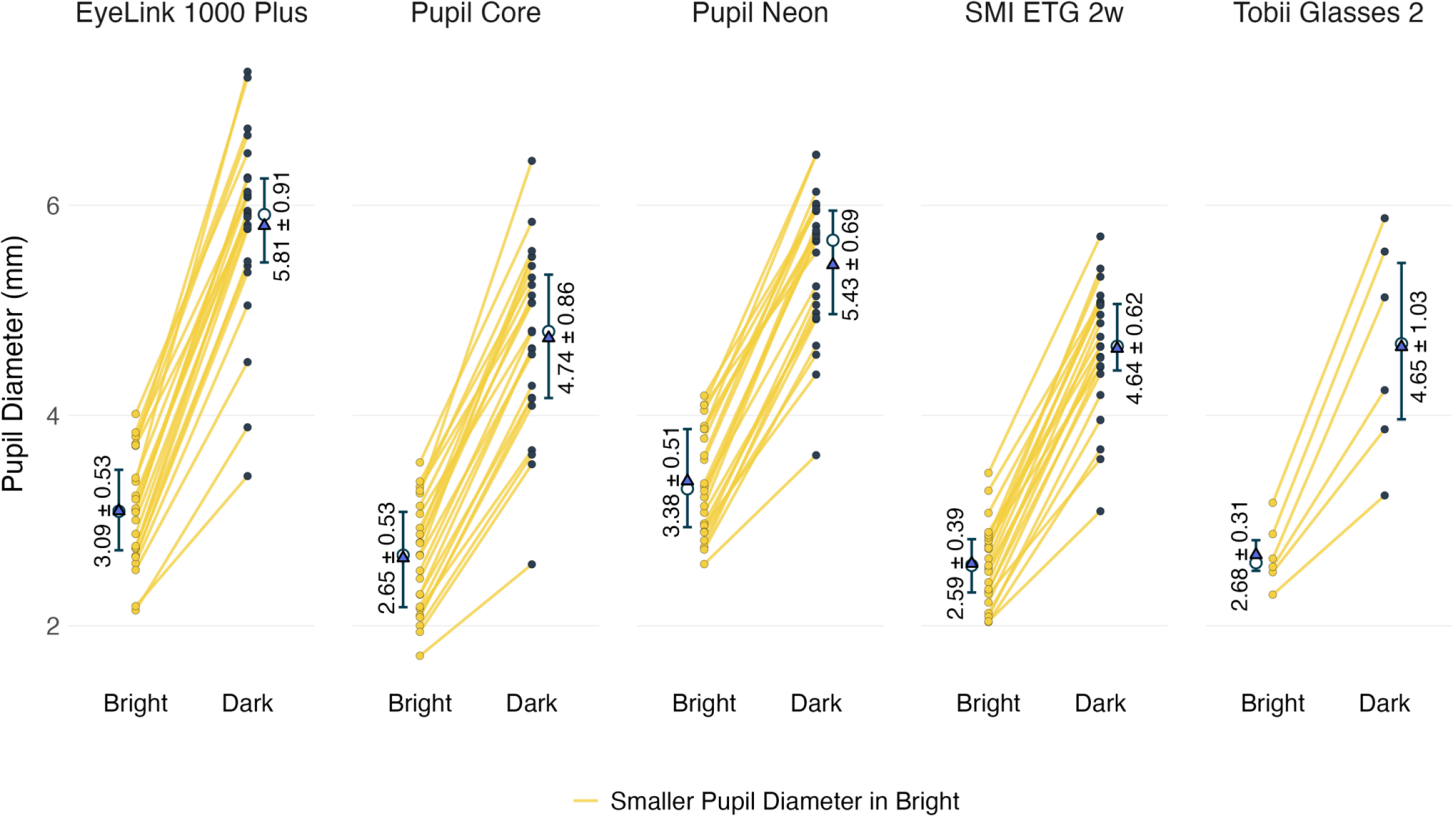
Pupil sizes during the bright and dark conditions across eye trackers. Each line represents an individual participant, where *yellow-colored lines* indicate participants who have smaller pupil diameter in bright conditions compared to dark conditions. *White circles * represent median and *blue triangles* represent mean values for each condition. The values in the plots indicate the mean ± standard deviation of pupil diameter measurements. *Error bars* are the interquartile range (IQR)

**Fig. 3:**
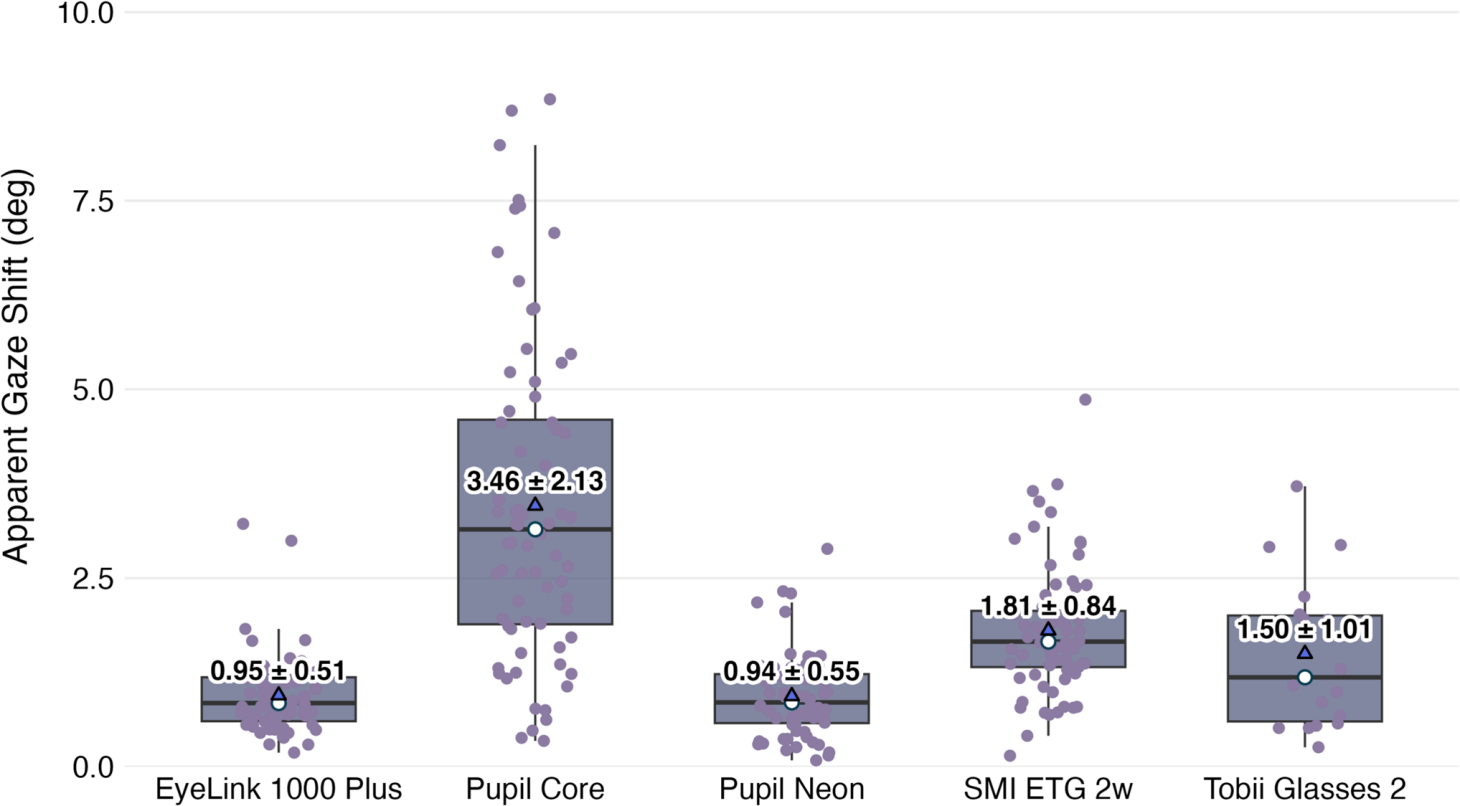
Apparent gaze shift across eye trackers. Each point represents an individual participant. *White circles *represent median and *blue triangles* represent mean values for each eye tracker. The values above each blue triangle indicate the mean ± standard deviation of apparent gaze shift measurements

The formulas used to compute these metrics are as follows:

##### Standard deviation (STD)

8 $$\begin{aligned} \text {STD} = \sqrt{\frac{\sum _{i=1}^{N} (x_i - \mu _x)^2 + (y_i - \mu _y)^2}{N}} \end{aligned}$$

where $(x_i, y_i)$ are the coordinates of each gaze point, $(\mu _x, \mu _y)$ is the mean gaze position, and *N* is the total number of gaze samples.

##### Root mean square sample-to-sample error (RMS-S2S)

9 $$\begin{aligned} \text {RMS-S2S} = \sqrt{\frac{\sum _{i=1}^{N-1} [(x_{i+1} - x_i)^2 + (y_{i+1} - y_i)^2]}{N-1}} \end{aligned}$$

Where $(x_i, y_i)$ are the coordinates of the current gaze sample, $(x_{i+1}, y_{i+1})$ are the coordinates of the subsequent gaze sample, and *N* is the total number of gaze samples.

### Data loss

In this paper, we operationalize data loss as the proportion of invalid samples during each trial. Invalid samples are identified as NaN (Not a Number) values reported directly by the eye trackers.

10 $$\begin{aligned} \text {Data Loss} = \frac{N_{\text {invalid}}}{N_{\text {total}}} \times 100\% \end{aligned}$$

where $N_{\text {invalid}}$ represents the number of samples with NaN values, and $N_{\text {total}}$ represents the total number of samples collected during the trial period before data cleaning procedures.

### Statistical analysis

We applied paired-sample *t* tests comparing each quality metric (accuracy, precision, apparent gaze shift) between bright and dark conditions for each eye tracker. To account for multiple comparisons, the resulting *p* values were Bonferroni-corrected. Statistical significance was determined at an adjusted threshold of $p < 0.05$. To quantify the magnitude of these effects, we calculated Cohen’s *d* effect sizes, which were interpreted as negligible ($d<0.2$), small ($0.2 \le d<0.5$), medium ($0.5 \le d < 0.8$), or large ($d \ge 0.8$). All statistical analyses were performed using R (version 4.4.2) with the tidyverse package (version 2.0.0).

## Results

### Pupil diameter

Pupil diameter measurements provided by each eye tracker revealed clear differences (Fig. 2) between the dark and bright conditions across all five devices, demonstrating the effectiveness of our method of using black and white screen backgrounds to manipulate pupil size in all of the participants. On average, the pupil diameter changed from about 2.88 mm in the bright condition to about 5.05 mm in the dark condition.

### Apparent gaze shift

First, we examine the shift in reported gaze direction under the bright and dark conditions. Figure 3 illustrates the apparent gaze shift exhibited by all eye trackers between the dark and bright conditions. All devices showed apparent gaze shifts significantly greater than zero, with the Pupil Core exhibiting the largest mean apparent gaze shift of $3.46^\circ$ ($t(23)=8.36, p<0.01$, Cohen’s $d=1.71$). In contrast, the EyeLink 1000 Plus and Pupil Neon showed the smallest mean apparent gaze shifts of $0.95^\circ$ ($t(23)=10.94, p<0.01$, Cohen’s $d=2.23$) and $0.94^\circ$ ($t(23)=10.76, p<0.01$, Cohen’s $d=2.20$), respectively. The SMI ETG 2w and Tobii Glasses 2 fell between these extremes, with mean apparent gaze shifts of $1.81^\circ$ ($t(23)=12.17, p<0.01$, Cohen’s $d=2.48$) and $1.49^\circ$ ($t(5)=4.06, p<0.05$, Cohen’s $d=1.66$), respectively.

### Accuracy

**Fig. 4:**
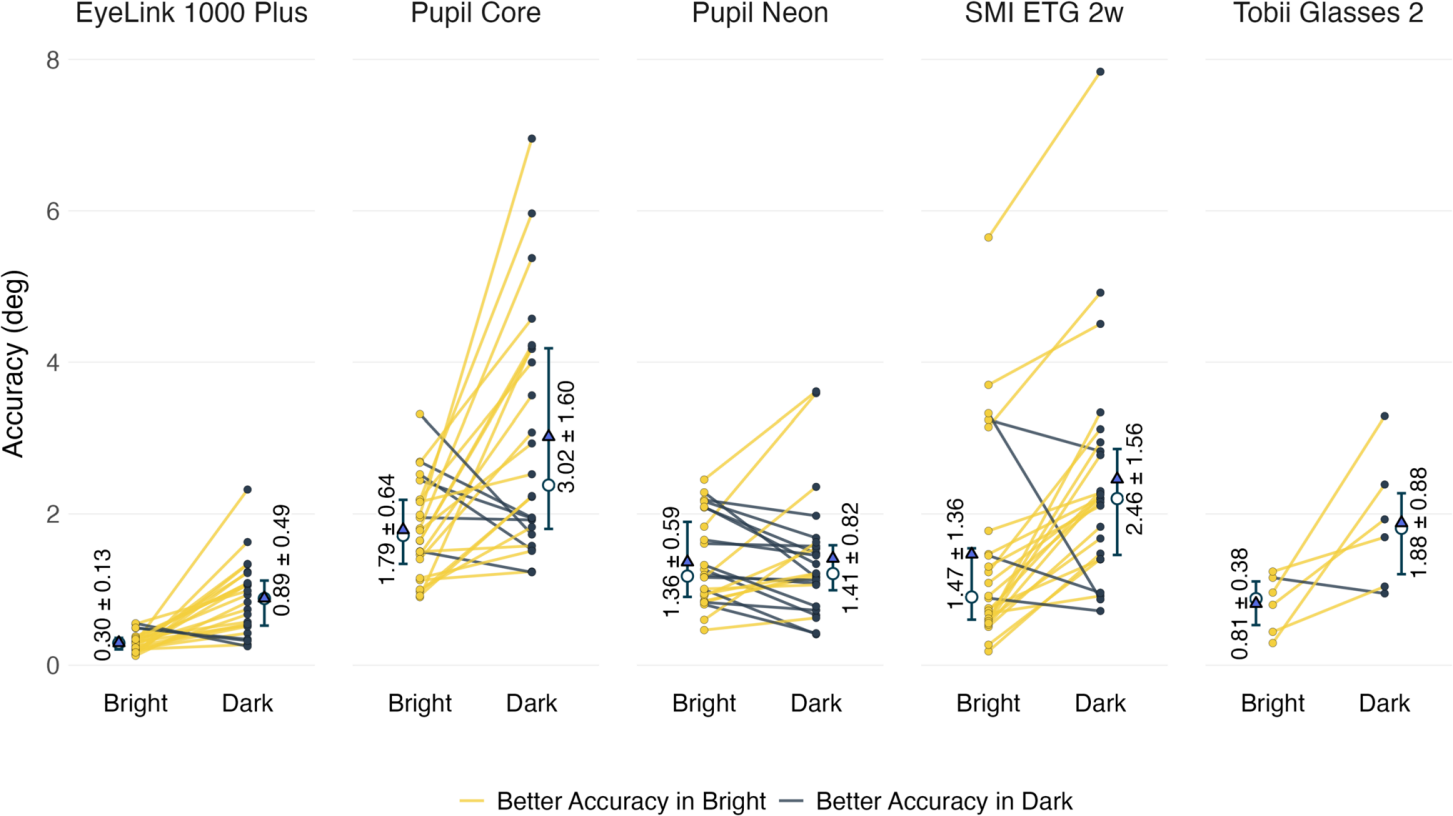
Comparison of participant-level accuracy changes between bright and dark conditions across eye trackers. Each line represents an individual participant, with *yellow lines* indicating better accuracy in bright conditions and *dark lines* indicating better accuracy in dark conditions. *White circles* represent median and *blue triangles* represent mean values for each condition. The values in the plots indicate the mean ± standard deviation of accuracy measurements. *Error bars* are the interquartile range (IQR)

Next, we examined the absolute accuracy of the eye trackers in the bright and dark conditions. Figure 4 reveals that across all systems, pupil size impacted accuracy for most participants. However, the direction and magnitude of these accuracy changes vary between participants. Overall, with the exception of the Pupil Neon ($t(23)=0.34, p=1.0$) and the Tobii Glasses 2 ($t(5)=2.65, p=0.23$), all eye trackers demonstrated significantly better accuracy in bright conditions compared to dark conditions ($p<0.01$, Cohen’s $d\ge 0.74$, see Table 2). The EyeLink 1000 Plus exhibited the best average accuracy at $0.29^\circ$ in the bright condition and $0.89^\circ$ in the dark condition, while the Pupil Core showed the worst average accuracy in both lighting conditions ($1.79^\circ$ in bright and $3.02^\circ$ in dark).

**Table 2:** Statistical analyses of accuracy between bright and dark conditions for each eye tracker

Eye tracker	Bright	Dark	*t* (*df*)	*p*	Adj. *p*	Cohen’s *d*
	Mean ± SD ($^{\circ }$)	Mean ± SD ($^{\circ }$)				
EyeLink 1000 Plus	0.30 ± 0.13	0.89 ± 0.49	5.31 (23)	< 0.01	< 0.01	1.08
Pupil Core	1.79 ± 0.64	3.02 ± 1.60	3.63 (23)	0.01	< 0.01	0.74
Pupil Neon	1.36 ± 0.59	1.41 ± 0.82	0.34 (23)	0.74	1.00	0.07
SMI ETG 2w	1.47 ± 1.36	2.46 ± 1.56	4.34 (23)	< 0.01	< 0.01	0.88
Tobii Glasses 2	0.81 ± 0.38	1.88 ± 0.88	2.65 (5)	0.05	0.29	1.08

### Precision

Do the different pupil sizes in the bright and dark conditions also affect the precision of the gaze signals produced by the eye trackers? To answer this question, we analyzed the STD and RMS-S2S precision.

**Fig. 5:**
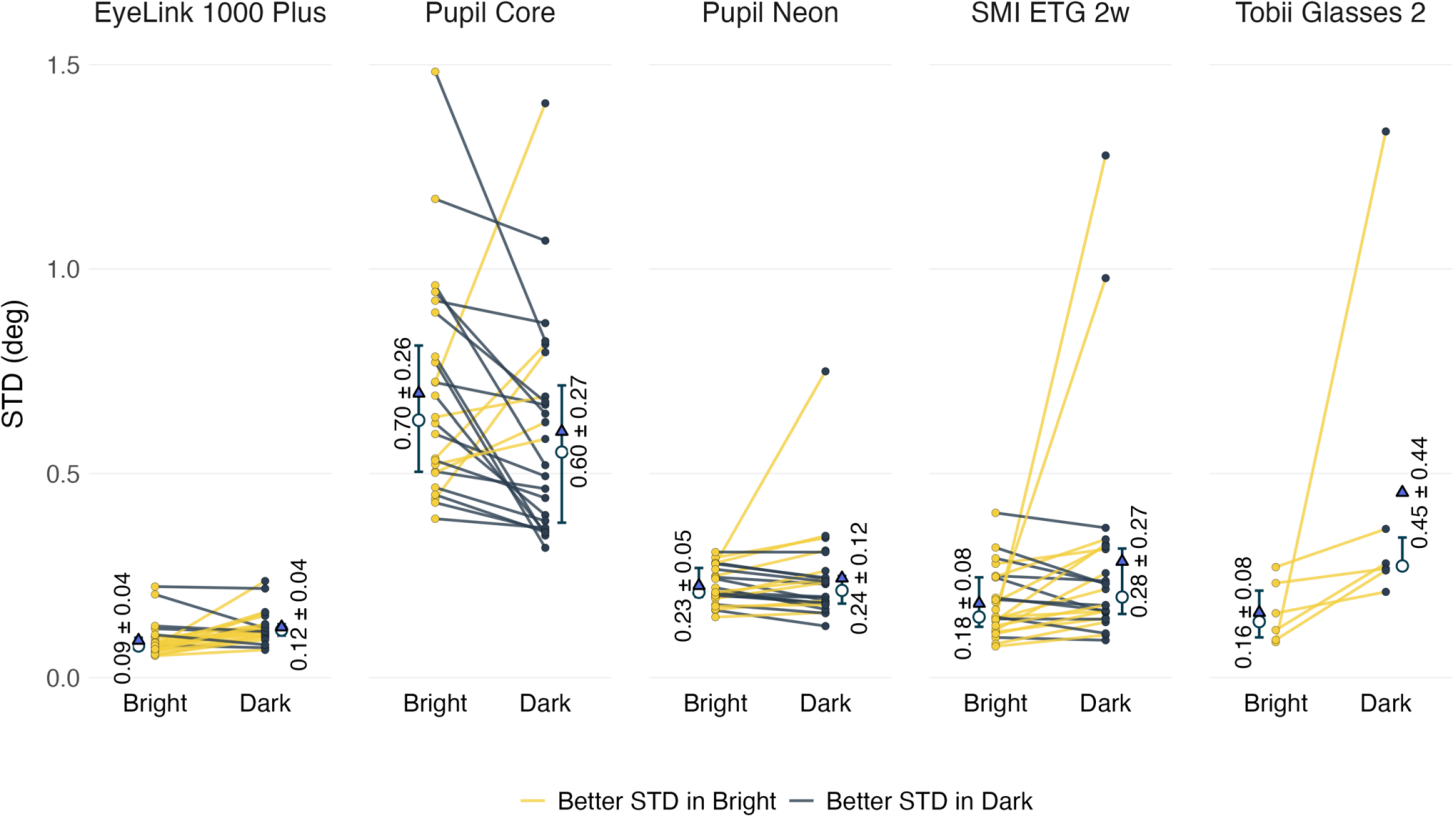
Comparison of participant-level STD changes between bright and dark conditions across eye trackers. Each line represents an individual participant, with *yellow lines* indicating better STD in bright conditions and *dark lines* indicating better STD in dark conditions. *White circles* represent median and *blue triangles* represent mean values for each condition. The values in the plots indicate the mean ± standard deviation of STD measurements. *Error bars* are the interquartile range (IQR)

Figure 5 shows the STD of eye tracker measurements under dark and bright conditions. For Tobii Glasses 2, all participants exhibited better STD in bright conditions; however, this difference was not statistically significant ($t(5)=1.53, p=0.93$). A similar trend was observed among most participants tested with the EyeLink 1000 Plus, and this effect was statistically significant ($t(23)=3.40, p=0.01$, Cohen’s $d= 0.69$). In contrast, the Pupil Core demonstrated better STD performance in dark conditions, though this difference was not statistically significant ($t(23)=-1.59, p=0.62$). No consistent pattern was observed for Pupil Neon ($t(23)=0.88$, $p=1.00$) or SMI ETG 2w ($t(23)=1.79$, $p=0.43$).

**Fig. 6:**
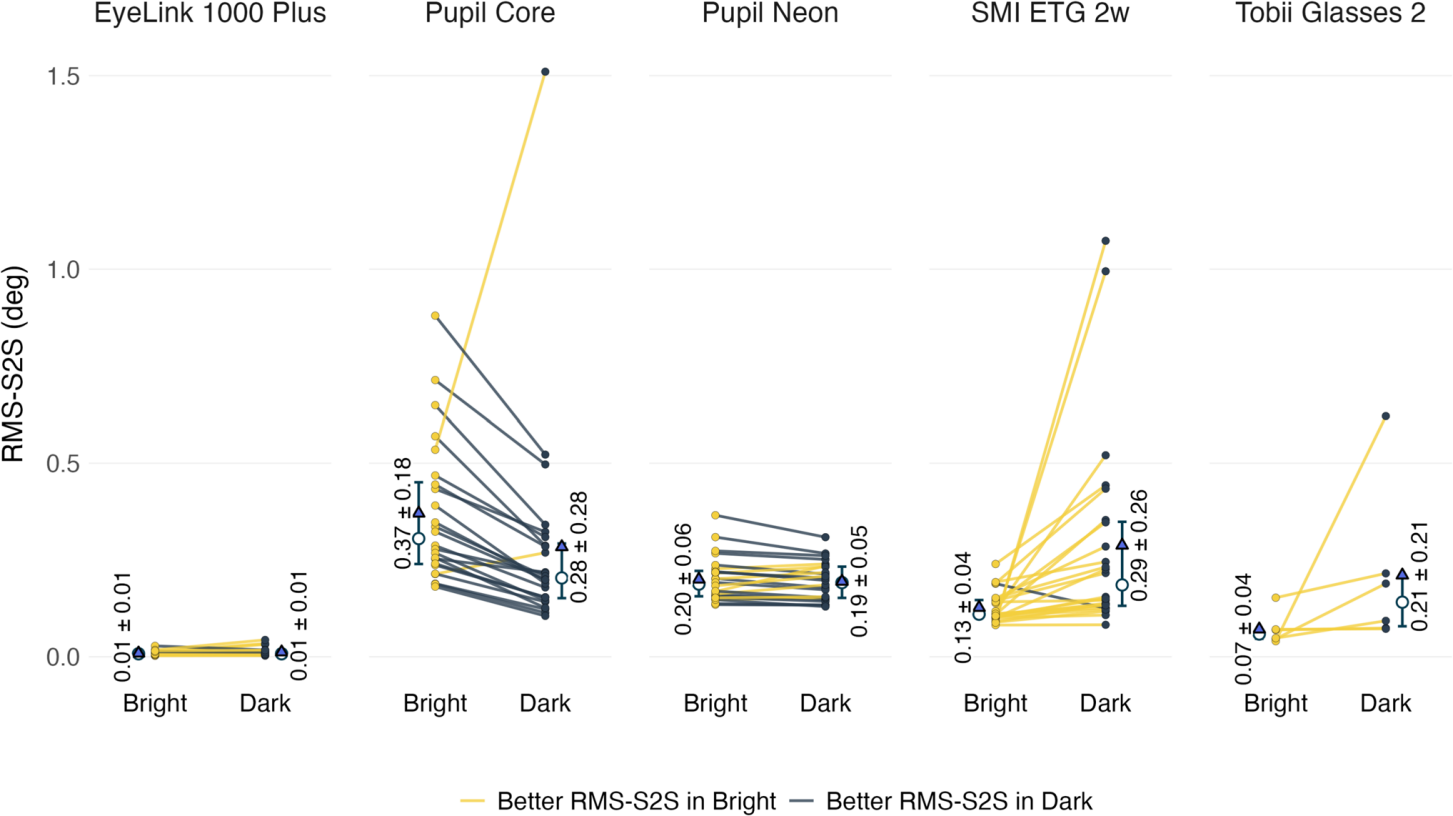
Comparison of participant-level RMS-S2S changes between bright and dark conditions across eye trackers. Each line represents an individual participant, with *yellow lines* indicating better RMS-S2S in bright conditions and *dark lines* indicating better RMS-S2S in dark conditions. *White circles* represent median and *blue triangles* represent mean values for each condition. The values in the plots indicate the mean ± standard deviation of RMS-S2S measurements. *Error bars* are the interquartile range (IQR)

Figure 6 shows the RMS-S2S of eye tracker measurements under dark and bright conditions. For Tobii Glasses 2, all participants exhibited better RMS-S2S in bright conditions; however, this difference was not statistically significant ($t(5)=1.54$, $p=0.92$). A similar trend was observed among most participants tested with SMI ETG 2w, and this difference was statistically significant ($t(23)=3.00$, $p=0.03$, Cohen’s $d=0.61$). For EyeLink 1000 Plus, the trend also favored bright conditions, but the result was not statistically significant ($t(23)=1.61$, $p=0.61$). In contrast, the Pupil Core demonstrated better RMS-S2S performance in dark conditions, though this was not statistically significant ($t(23)=-1.74$, $p=0.47$, Cohen’s $d=-0.36$). No consistent pattern was observed for Pupil Neon ($t(23)=-1.10$, $p=1.00$).

### Data loss

**Fig. 7:**
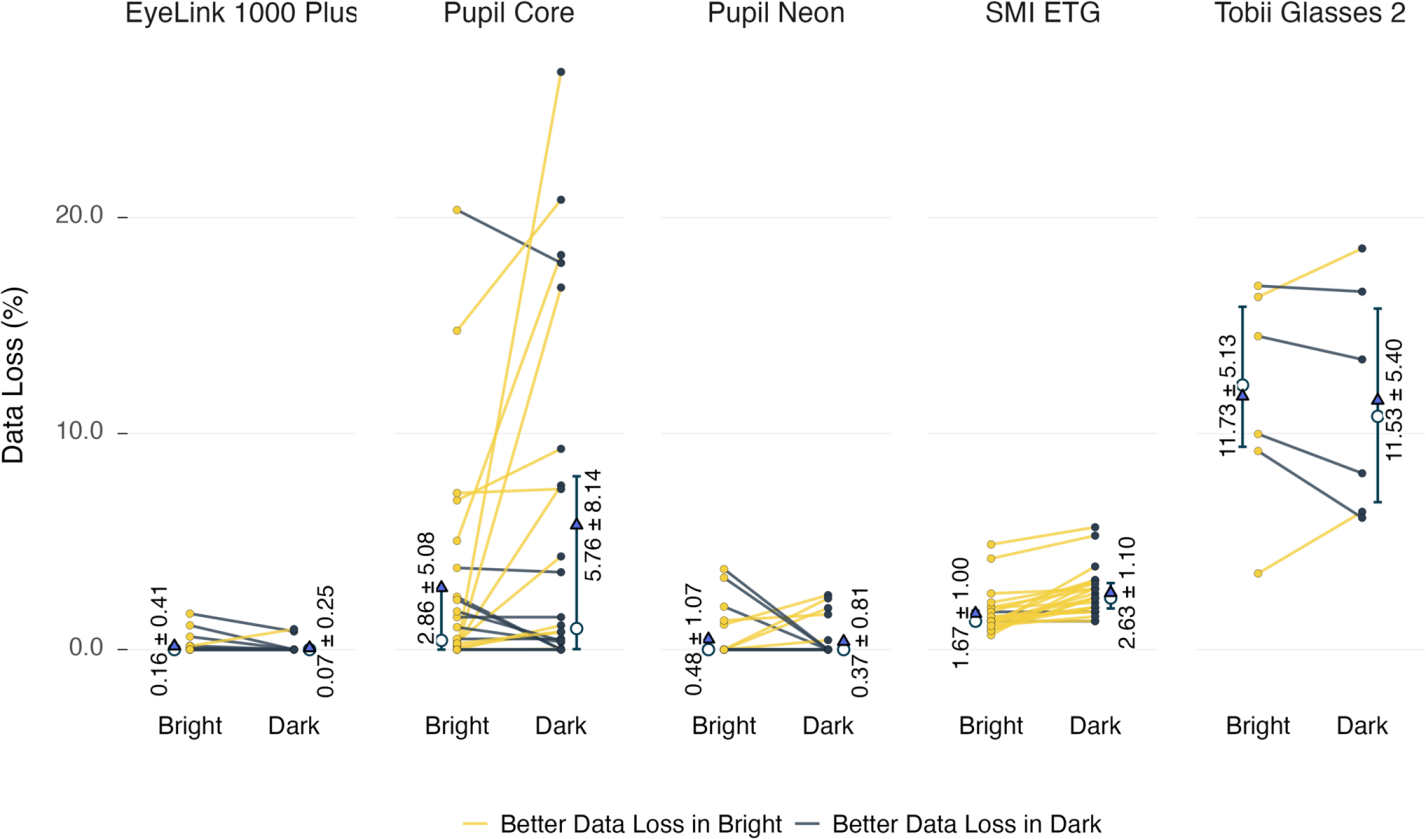
Comparison of participant-level data loss changes between bright and dark conditions across eye trackers. Each line represents an individual participant, with *yellow lines* indicating better data loss in bright conditions and *dark lines* indicating better data loss in dark conditions. *White circles* represent median and *blue triangles* represent mean values for each condition. The values in the plots indicate the mean ± standard deviation of data loss measurements. *Error bars* are the interquartile range (IQR)

Finally, we investigated whether lighting conditions influenced data loss across the different eye trackers. Figure 7 shows the participant-level changes in data loss between bright and dark conditions across all eye trackers. Among all eye trackers, only the SMI ETG 2w showed a statistically significant difference in data loss between lighting conditions ($t(23)=6.00$, $p<0.01$, Cohen’s $d=1.23$). In contrast, none of the other devices demonstrated significant differences in data loss between conditions ($p \ge 0.05$). Both the EyeLink 1000 Plus and Pupil Neon had median data loss values of $0\%$ for both lighting conditions, indicating that most participants experienced no data loss with these systems. The Pupil Core showed notable outliers, with maximum data loss values of $20.4\%$ in bright and $26.7\%$ in dark conditions.

## Discussion

In this paper, we have addressed three research questions: Do head-mounted eye trackers exhibit PSA?; do pupil size changes also influence accuracy, precision, and data loss in head-mounted eye trackers?; and do appearance-based head-mounted eye trackers also exhibit PSA? By investigating the effects of pupil size variations on data quality across four head-mounted eye trackers and one desktop reference system, our findings reveal that pupil size artifacts are present in all head-mounted systems tested, including both traditional and appearance-based methods. Furthermore, changes in pupil size significantly affect data quality metrics such as accuracy, precision, and data loss.

The EyeLink 1000 Plus showed an apparent gaze shift consistent with prior reports (Drewes et al., 2014; Hooge et al., 2021; Ivanov & Blanche, 2011), confirming our protocol reliably induces PSA. All tested eye trackers showed statistically significant ($p<0.01$) apparent gaze shift with large effect size (Cohen’s $d \ge 1.66$), indicating that PSA exists in head-mounted eye trackers regardless of their technologies. Across devices, we observed substantial variation in PSA magnitudes: the EyeLink showed a mean apparent gaze shift of 0.95$^\circ$, closely matched by the Pupil Neon at 0.94$^\circ$. Other head-mounted trackers exhibited larger shifts: Tobii Pro Glasses 2 at 1.49$^\circ$, SMI ETG 2w at 1.81$^\circ$, and Pupil Core at 3.46$^\circ$. This finding builds upon previous research conducted with desktop-based eye trackers (e.g., Drewes et al., 2012; Hooge et al., 2021; Wyatt, 2010) and extends it to head-mounted systems.

Notably, although the EyeLink maintained the best absolute accuracy (0.29$^\circ$ bright, 0.89$^\circ$ dark), its PSA magnitude was nearly identical to the Pupil Neon despite the Neon’s lower accuracy (1.36$^\circ$ bright, 1.41$^\circ$ dark). This indicates that PSA magnitude and overall accuracy are not directly correlated; a tracker can have moderate accuracy with low PSA magnitude (Pupil Neon) or poorer accuracy with high PSA magnitude (Pupil Core: 1.79$^\circ$ bright, 3.02$^\circ$ dark accuracy with 3.46$^\circ$ PSA). The finding that an appearance-based system without corneal reflections (Pupil Neon) matched the P-CR-based EyeLink’s PSA performance suggests that different technical approaches can achieve similar robustness to pupil-size changes. However, as these devices are proprietary and closed source, the specific implementation details affecting PSA magnitude cannot be examined.

Regarding our second research question, we found that pupil size changes indeed influence other data quality measures in head-mounted eye trackers. With the exception of the Pupil Neon, all tested devices exhibited a decrease in accuracy under dark pupil conditions compared to bright conditions. However, it would be incorrect to conclude that bright conditions inherently provide better accuracy. Work by Salari and Bednarik (2024) (using SMI ETG 2w) suggests that eye trackers usually perform better in lighting conditions that match those used during calibration. Since all our calibrations were performed in bright conditions, this methodological choice likely accounts for the observed accuracy advantage in bright testing conditions.

It is noteworthy that the accuracy for the Pupil Neon was also affected by the lighting condition for most participants, but that the direction of the effect varied between participants. Specifically, while the accuracy was worse in the bright condition compared to the dark condition for some participants, for others the effect was in the opposite direction with better accuracy in the bright condition. This inconsistency in accuracy performance among participants in Pupil Neon contrasts with the consistent accuracy decline observed in all other eye trackers under dark conditions. This difference may suggest that the training set used for creating the device’s gaze estimation pipeline was crafted with some consideration to PSA, leading to relatively small gaze shifts and inconsistent effects of pupil size on overall accuracy. However, we cannot rule out that inadequate offset correction during data recording, given the rudimentary interface provided for this in the Pupil Companion app, may instead have caused the variability in accuracy performance.

The effects of pupil size on precision varied considerably across eye trackers, with some devices (Tobii Glasses 2, SMI ETG 2w) showing better precision under bright conditions, while others (Pupil Core) performed better in dark conditions. This finding suggests that optimal precision performance is device-dependent rather than universally tied to specific pupil size condition.

Data loss rates remained largely unaffected by lighting conditions across most eye trackers, with only the SMI ETG 2w demonstrating a statistically significant difference between bright and dark conditions. This indicates that pupil size variations have minimal impact on data capture reliability for most head-mounted eye trackers.

Regarding the third research question on appearance-based systems, the Pupil Neon, which employs end-to-end deep learning for gaze estimation rather than traditional pupil feature detection, still exhibited significant PSA. It showed the smallest apparent gaze shift ($0.94^\circ$) among the tested head-mounted eye trackers. This suggests that appearance-based methods have the potential to mitigate, though not entirely eliminate, PSA. As noted above, while we can only guess, likely the manufacturer has ensured to include a range of pupil sizes in their training set in an effort to reduce PSA. As this effect is likely device- and training set-dependent, it remains to be seen whether other appearance-based head-mounted eye trackers also exhibit decreased PSA.

### Practical implications

All tested eye trackers, regardless of their underlying gaze estimation methods, were affected by PSA. This highlights PSA as a common issue across eye trackers that should be accounted for in the study design and analysis. Based on our findings, we offer the following recommendations:

1. *Awareness of data quality variations*: Researchers should be mindful that PSA causes substantial apparent gaze shifts in head-mounted eye trackers. The magnitude of apparent gaze shifts can vary widely depending on the device, ranging for example, from under $1^\circ$ in some systems (e.g., Pupil Neon) to over $3^\circ$ in others (e.g., Pupil Core). The device-specific nature of PSA means that researchers should test their selected eye trackers under the expected illumination conditions of their study to verify that, even with PSA effects, the accuracy remains within an acceptable range for their specific research questions and study design. We further recommend conducting calibration or offset correction under illumination conditions as similar as possible to those during actual data recordings.

2. *Documenting conditions*: Researchers should document not only the eye tracker used but also the calibration conditions, testing environment illumination, and observed pupil size ranges (*cf.* Dunn et al., 2023). The substantial individual differences observed in our participant-level accuracy data (*cf.* Figure 4) reveal that illumination changes can improve accuracy for some participants while decreasing it for others and reporting these methodological details would allow better interpretation of the results.

### Limitations and future directions

While our study provides comprehensive insights into PSA across head-mounted eye trackers, several limitations should be acknowledged. First, we tested only two illumination conditions to manipulate pupil size. Although this allowed us to demonstrate the existence and magnitude of PSA at two extremes, it does not capture the full range of pupil diameters encountered in real-world settings. Future work could expand on this by sampling a broader range of pupil sizes to examine the continuity of PSA effects and whether the relationship between pupil size and gaze error is linear or follows the more complex pattern often observed in studies with desktop-mounted eye trackers (e.g., Hooge et al., 2021).

Second, all calibrations were conducted under bright conditions. While this approach ensured consistency across all the eye trackers tested in this study, it remains unknown whether calibrating under darker conditions would yield similar PSA patterns, but shifted such that the best accuracy is achieved during dark conditions. Investigating the role of illumination during calibration could help determine whether PSA characteristics are primarily driven by relative changes in pupil size or also shaped by the absolute conditions at calibration. As such, future work should test whether calibration on a dark background (for the devices where this is possible) would lead to PSA-shifts in the opposite direction as calibration on a bright background.

Third, we used only one appearance-based eye tracker in our comparison. While this allowed us to examine PSA characteristics in this category of eye trackers, future studies should investigate other appearance-based eye trackers to determine whether the PSA patterns observed here are generalizable across different appearance-based gaze estimation methods.

Fourth, with our dataset, we cannot study the underlying mechanisms of PSA. Choe et al. (2016) proposed that PSA can be described by two components: (a) an idiosyncratic component, reflecting physiological pupil-center shifts where changes in pupil size move the geometric pupil center rather than scaling concentrically, thereby biasing pupil-based gaze estimates (Mathur et al., 2014; Wyatt, 1995; Wildenmann & Schaeffel, 2013); and (b) a viewing-direction-dependent component, which varies systematically with eye-tracker geometry and camera orientation. As mentioned in the Introduction, Hooge et al. (2021) confirmed the presence of both components across different devices and over time, and showed that PSA magnitude varies systematically with viewing direction. Their simulation analyses further indicated that the viewing-direction component cannot be fully explained by corneal optics, leaving open the possibility that sensor geometry or algorithmic factors also may contribute. To evaluate these mechanisms directly, access to the eye tracker’s internal signals and parameters is required. Access to pupil center and corneal reflection positions is essential for testing these mechanisms, as they are required to calculate pupil-center shifts. Raw eye images are also necessary to identify and correct potential measurement errors in pupil-center and corneal reflection detection introduced by the device’s internal image-processing algorithms, and can be used to calculate pupil-center shifts independently when no corneal reflection is available. Camera–eye geometry and calibration parameters are necessary to test the underlying mechanisms, as the magnitude of PSA has been shown by Hooge et al. (2021) to depend on the participant’s viewing direction relative to the orientation of the camera, with systematic variation across viewing directions. Knowledge of system geometry parameters (e.g., camera optics and position, IR light-source positions, nodal point assumptions) is needed to determine the extent to which the geometry of the eye tracker (the relative positions and orientations of the eye and camera) contributes to PSA magnitude in each eye tracker.

Algorithmic transparency is needed to understand the impact of the computational model used by each device on the observed PSA. Whether an eye tracker applies the pupil–CR method, higher-order mappings, 3D model fitting, or appearance-based methods may contribute to how PSA manifests in that device. These parameters, especially the gaze-estimation algorithms used by each device, are not provided by the closed-source software of most of the eye trackers tested in this study. We therefore encourage future studies to employ open hardware and software device that provide access to raw images, feature coordinates, and camera–eye geometry parameters, enabling direct tests of the hypothesized physiological and geometric origins of PSA.

## Conclusion

Our study demonstrates that head-mounted video-based eye trackers exhibit PSA just like their desktop-mounted counterparts. The magnitude of PSA, as well as the effect of pupil size change on other data quality measures, differed between devices. The finding that a device that uses an appearance-based method (Pupil Neon) showed less sensitivity to pupil size variations offers promising directions for future eye tracker development. Given the growing use of head-mounted eye trackers in real-world research, understanding and accounting for PSA is essential for ensuring the validity and reliability of gaze data. Researchers employing these systems should be aware of the potential impact of pupil size variations on measurement accuracy and consider appropriate control or compensation strategies based on their specific research requirements.

## Data Availability

The dataset generated during the current study is available in https://github.com/mh-salari/psa_data_quality/tree/main/data.

## Footnotes

1 https://github.com/mh-salari/zarafe

2 For the recommended procedure for pupil-size calibration and conversion to millimeters for the EyeLink, see SR Research FAQ “How can I convert pupil size to mm?” (login required): https://www.sr-research.com/support/thread-154.html; or the public tutorial video: https://www.youtube.com/watch?v=PfVHvqch0ME

## Code availability

The analysis code used in this study is available on GitHub https://github.com/mh-salari/psa_data_quality. The custom annotation tool used in this study is available at https://github.com/mh-salari/zarafe.

## Declarations

### Open Practices Statement

This study follows open science practices. Processed data, analysis code, and all materials used in this study have been made publicly available at https://github.com/mh-salari/psa_data_quality.

## References

[CR1] Andersson, R., Larsson, L., Holmqvist, K., Stridh, M., & Nystöm, M. (2017). One algorithm to rule them all? An evaluation and discussion of ten eye movement event-detection algorithms. *Behavior Research Methods,**49*(2), 616–637. 10.3758/s13428-016-0738-9 27193160 10.3758/s13428-016-0738-9

[CR2] Aziz, S., & Komogortsev, O. (2022). An assessment of the eye tracking signal quality captured in the HoloLens 2. (2022). *Symposium on Eye Tracking Research and Applications* (pp. 1–6). Seattle WA USA: ACM.

[CR3] Beatty, J. (1982). Task-evoked pupillary responses, processing load, and the structure of processing resources. *Psychological Bulletin*, *91*(2), 276–292, 10.1037/0033-2909.91.2.276 7071262

[CR4] Cheng, Y., Wang, H., Bao, Y., & Lu, F. (2024). Appearance-based gaze estimation with deep learning: A review and benchmark. *IEEE Transactions on Pattern Analysis and Machine Intelligence*, *46*(12), 7509–7528, 10.1109/TPAMI.2024.3393571 10.1109/TPAMI.2024.3393571 38662567

[CR5] Choe, K. W., Blake, R., & Lee, S.-H. (2016). Pupil size dynamics during fixation impact the accuracy and precision of video-based gaze estimation. *Vision Research,**118*, 48–59. 10.1016/j.visres.2014.12.018 25578924 10.1016/j.visres.2014.12.018

[CR6] Dik, V. K., Hooge, I. T. C., Van Oijen, M. G., & Siersema, P. D. (2016). Measuring gaze patterns during colonoscopy: A useful tool to evaluate colon inspection? *European Journal of Gastroenterology and Hepatology,**28*(12), 1400–1406. 10.1097/MEG.0000000000000717 27769078 10.1097/MEG.0000000000000717

[CR7] Drewes, J., Masson, G. S., & Montagnini, A. (2012). Shifts in reported gaze position due to changes in pupil size: Ground truth and compensation. Proceedings of the Symposium on Eye Tracking Research and Applications (pp. 209–212). New York, NY, USA: Association for Computing Machinery.

[CR8] Drewes, J., Zhu, W., Hu, Y., & Hu, X. (2014). Smaller is better: Drift in gaze measurements due to pupil dynamics. *PLOS One*, *9*(10), e111197, 10.1371/journal.pone.0111197 10.1371/journal.pone.0111197 PMC4206464 25338168

[CR9] Duchowski, A. T. (2017). *Eye tracking methodology*. Cham: Springer International Publishing.

[CR10] Dunn, M.J., Alexander, R.G., Amiebenomo, O.M., Arblaster, G., Atan, D., Erichsen, J.T., ..., Sprenger, A. (2023). Minimal reporting guideline for research involving eye tracking (2023 edition). Behavior Research Methods, 56 (5), 4351– 4357. 10.3758/s13428-023-02187-1 10.3758/s13428-023-02187-1 PMC11225961 37507649

[CR11] Ehinger, B. V., Groß, K., Ibs, I., & König, P. (2019). A new comprehensive eye-tracking test battery concurrently evaluating the pupil labs glasses and the EyeLink 1000. *PeerJ,**7*, Article e7086. 10.7717/peerj.7086 31328028 10.7717/peerj.7086 PMC6625505

[CR12] Fairbairn, D., & Hepburn, J. (2023). Eye-tracking in map use, map user and map usability research: What are we looking for? *International Journal of Cartography,**9*(2), 231–254. 10.1080/23729333.2023.2189064

[CR13] Hansen, D., & Ji, Q. (2010). In the eye of the beholder: A survey of models for eyes and gaze. *IEEE Transactions on Pattern Analysis and Machine Intelligence,**32*(3), 478–500. 10.1109/TPAMI.2009.30 20075473 10.1109/TPAMI.2009.30

[CR14] Holmqvist, K., & Andersson, R. (2017). *Eye tracking: A comprehensive guide to methods, paradigms, and measures (2nd* (edition). Lund, Sweden: Lund EyeTracking Research Institute.

[CR15] Holmqvist, K., Nyström, M., Mulvey, F. (2012). Eye tracker data quality: What it is and how to measure it. Proceedings of the Symposium on Eye Tracking Research and Applications (pp. 45–52). Santa Barbara California: ACM.

[CR16] Hooge, I. T. C., Hessels, R. S., Niehorster, D. C., Andersson, R., Skrok, M. K., Konklewski, R., ..., & Nyström, M. (2024). Eye tracker calibration: How well can humans refixate a target? *Behavior Research Methods,**57*(1), 23. 10.3758/s13428-024-02564-4 10.3758/s13428-024-02564-4 PMC11659352 39702537

[CR17] Hooge, I. T. C., Hessels, R. S., & Nyström, M. (2019). Do pupil-based binocular video eye trackers reliably measure vergence? *Vision Research,**156*, 1–9. 10.1016/j.visres.2019.01.004 30641092 10.1016/j.visres.2019.01.004

[CR18] Hooge, I. T. C., Niehorster, D. C., Hessels, R. S., Benjamins, J. S., & Nyström, M. (2022). How robust are wearable eye trackers to slow and fast head and body movements? *Behavior Research Methods,**55*(8), 4128–4142. 10.3758/s13428-022-02010-3 36326998 10.3758/s13428-022-02010-3 PMC10700439

[CR19] Hooge, I. T. C., Niehorster, D. C., Hessels, R. S., Cleveland, D., & Nyström, M. (2021). The pupil-size artefact (PSA) across time, viewing direction, and different eye trackers. *Behavior Research Methods,**53*(5), 1986–2006. 10.3758/s13428-020-01512-2 33709298 10.3758/s13428-020-01512-2 PMC8516786

[CR20] Huckauf, A. (2018). Systematic shifts of fixation disparity accompanying brightness changes. *Proceedings of the 2018 ACM Symposium on Eye Tracking Research & Applications* (pp. 1–5). Warsaw Poland: ACM.

[CR21] Ivanov, P., & Blanche, T. (2011). Improving gaze accuracy and predicting fixation in real time with video based eye trackers. *Journal of Vision,**11*(11), 505–505. 10.1167/11.11.505

[CR22] Jongerius, C., Callemein, T., Goedemé, T., Van Beeck, K., Romijn, J. A., Smets, E. M. A., & Hillen, M. A. (2021). Eye-tracking glasses in face-to-face interactions: Manual versus automated assessment of areas-of-interest. *Behavior Research Methods,**53*(5), 2037–2048. 10.3758/s13428-021-01544-2 10.3758/s13428-021-01544-2 PMC8516759 33742418

[CR23] Kiefer, P., Giannopoulos, I., & Raubal, M. (2013). Using eye movements to recognize activities on cartographic maps. Proceedings of the 21st ACM SIGSPATIAL International Conference on Advances in Geographic Information Systems (pp. 488–491). Orlando Florida: ACM.

[CR24] Laeng, B., Sirois, S., & Gredebäck, G. (2012). Pupillometry: A window to the preconscious? Perspectives on Psychological Science, 7 (1), 18–27, 10.1177/1745691611427305 10.1177/1745691611427305 26168419

[CR25] Larrazabal, A., García Cena, C., & Martínez, C. (2019). Video-oculography eye tracking towards clinical applications: A review. *Computers in Biology and Medicine,**108*, 57–66. 10.1016/j.compbiomed.2019.03.025 31003180 10.1016/j.compbiomed.2019.03.025

[CR26] Macinnes, J. J., Iqbal, S., Pearson, J., & Johnson, E. N. (2018). Wearable eyetracking for research: Automated dynamic gaze mapping and accuracy/precision comparisons across devices. bioRxiv.

[CR27] Mathôt, S. (2018). Pupillometry: Psychology, physiology, and function. *Journal of Cognition,**1*(1), 16. 10.5334/joc.18 31517190 10.5334/joc.18 PMC6634360

[CR28] Mathur, A., Gehrmann, J., & Atchison, D. A. (2014). Influences of luminance and accommodation stimuli on pupil size and pupil center location. *Investigative Opthalmology & Visual Science,**55*(4), 2166. 10.1167/iovs.13-13492 10.1167/iovs.13-13492 24595386

[CR29] Merchant, J., Morrissette, R., Porterfield, J.L. (1974). Remote measurement of eye direction allowing subject motion over one cubic foot of space. *IEEE Transactions on Bio-Medical Engineering*, *BME-21*(4), 309–317, 10.1109/TBME.1974.324318 10.1109/TBME.1974.324318 4837476

[CR30] Niehorster, D. C., Hessels, R. S., Benjamins, J. S., Nyström, M., & Hooge, I. T. C. (2023). GlassesValidator: A data quality tool for eye tracking glasses. *Behavior Research Methods,**56*(3), 1476–1484. 10.3758/s13428-023-02105-5 10.3758/s13428-023-02105-5 PMC10991001 37326770

[CR31] Niehorster, D. C., Santini, T., Hessels, R. S., Hooge, I. T. C., Kasneci, E., & Nyström, M. (2020). The impact of slippage on the data quality of head-worn eye trackers. *Behavior Research Methods,**52*(3), 1140–1160. 10.3758/s13428-019-01307-0 31898290 10.3758/s13428-019-01307-0 PMC7280360

[CR32] Niehorster, D.C., Zemblys, R., Beelders, T., Holmqvist, K. (2020, December). Characterizing gaze position signals and synthesizing noise during fixations in eye-tracking data. *Behavior Research Methods*, *52*(6), 2515–2534, 10.3758/s13428-020-01400-9 10.3758/s13428-020-01400-9 PMC7725698 32472501

[CR33] Nyström, M., Hooge, I. T. C., Hessels, R. S., Andersson, R., Hansen, D. W., Johansson, R., & Niehorster, D. C. (2025). The fundamentals of eye tracking part 3: How to choose an eye tracker. *Behavior Research Methods,**57*(2), 67. 10.3758/s13428-024-02587-x 39843609 10.3758/s13428-024-02587-x PMC11754381

[CR34] Orquin, J. L., & Holmqvist, K. (2018). Threats to the validity of eye-movement research in psychology. *Behavior Research Methods,**50*(4), 1645–1656. 10.3758/s13428-017-0998-z 29218588 10.3758/s13428-017-0998-z

[CR35] Pastel, S., Chen, C.-H., Martin, L., Naujoks, M., Petri, K., & Witte, K. (2021). Comparison of gaze accuracy and precision in real-world and virtual reality. *Virtual Reality,**25*(1), 175–189. 10.1007/s10055-020-00449-3

[CR36] Pupil Labs (2024a). Pupil Core eye tracking platform technical specifications. Retrieved 2024-07-25, from https://pupil-labs.com/products/core/tech-specs

[CR37] Pupil Labs (2024b). Pupil Neon eye tracking module technical specifications. Retrieved 2024-07-25, from https://pupil-labs.com/products/neon/specs

[CR38] Rogers, S. L., Speelman, C. P., Guidetti, O., & Longmuir, M. (2018). Using dual eye tracking to uncover personal gaze patterns during social interaction. *Scientific Reports,**8*(1), 4271. 10.1038/s41598-018-22726-7 29523822 10.1038/s41598-018-22726-7 PMC5844880

[CR39] Salari, M., & Bednarik, R. (2024). Investigating the impact of illumination change on the accuracy of head-mounted eye trackers: A protocol and initial results. Companion Proceedings of the 26th International Conference on Multimodal Interaction (pp. 204–210). San Jose Costa Rica: ACM.

[CR40] SensoMotoric Instruments (SMI) (2024). SMI eye tracking glasses 2 wireless technical specifications. Retrieved 2024-07-25, from https://www.dpg.unipd.it/sites/dpg.unipd.it/files/smi prod ETG 120Hz asgm%20%281%29.pdf (Archived version available at https://web.archive.org/web/20250225203713/https://www .dpg.unipd.it/sites/dpg.unipd.it/files/smi prod ETG 120Hz asgm%20(1).pdf)

[CR41] SR Research Ltd (2024). EyeLink 1000 plus technical specifications. Retrieved 2024-07-25, from https://www.sr-research.com/wp-content/uploads/ 2017/11/eyelink-1000-plus-specifications.pdf (Archived version available at https://web.archive.org/web/20250506122810/https://www.sr-research .com/wp-content/uploads/2017/11/eyelink-1000-plus-specifications.pdf)

[CR42] Startsev, M., & Zemblys, R. (2022). Evaluating eye movement event detection: A review of the state of the art. *Behavior Research Methods,**55*(4), 1653–1714. 10.3758/s13428-021-01763-7 35715615 10.3758/s13428-021-01763-7

[CR43] Stein, N., Niehorster, D. C., Watson, T., Steinicke, F., Rifai, K., Wahl, S., & Lappe, M. (2021). A comparison of eye tracking latencies among several commercial head-mounted displays. *I-perception,**12*(1), 2041669520983338. 10.1177/2041669520983338 33628410 10.1177/2041669520983338 PMC7883159

[CR44] Stuart, S., Alcock, L., Godfrey, A., Lord, S., Rochester, L., & Galna, B. (2016). Accuracy and re-test reliability of mobile eye-tracking in parkinson’s disease and older adults. *Medical Engineering & Physics,**38*(3), 308–315. 10.1016/j.medengphy.2015.12.001 26786676 10.1016/j.medengphy.2015.12.001

[CR45] Thaler, L., Schütz, A., Goodale, M., & Gegenfurtner, K. (2013). What is the best fixation target? The effect of target shape on stability of fixational eye movements. *Vision Research,**76*, 31–42. 10.1016/j.visres.2012.10.012 23099046 10.1016/j.visres.2012.10.012

[CR46] Tobii. (2024). Tobii Pro Glasses 2 technical specifications. Retrieved 2024-07-25, from https://nbtltd.com/wp-content/uploads/2018/05/ tobiiproproductdescription.pdf (Archived version available at https://web.archive.org/web/20241210231233/https://nbtltd.com/wp-content/uploads/2018/05/tobiiproproductdescription.pdf)

[CR47] Tonsen, M., Baumann, C. K., & Dierkes, K. (2020). A high-level description and performance evaluation of pupil invisible (No. arXiv:2009.00508). arXiv.

[CR48] Vehlen, A., Standard, W., & Domes, G. (2022). How to choose the size of facial areas of interest in interactive eye tracking. *PLOS One,**17*(2), Article e0263594. 10.1371/journal.pone.0263594 10.1371/journal.pone.0263594 PMC8815978 35120188

[CR49] Vrzakova, H., & Bednarik, R. (2012). Hard lessons learned: Mobile eyetracking in cockpits. Proceedings of the 4th Workshop on Eye Gaze in Intelligent Human Machine Interaction (pp. 1–6). Santa Monica California: ACM.

[CR50] Wildenmann, U., & Schaeffel, F. (2013). Variations of pupil centration and their effects on video eye tracking. *Ophthalmic and Physiological Optics,**33*(6), 634–641. 10.1111/opo.12086 24102513 10.1111/opo.12086

[CR51] Wyatt, H. J. (1995). The form of the human pupil. *Vision Research,**35*(14), 2021–2036. 10.1016/0042-6989(94)00268-Q 10.1016/0042-6989(94)00268-q 7660606

[CR52] Wyatt, H. J. (2010). The human pupil and the use of video-based eyetrackers. *Vision Research,**50*(19), 1982–1988. 10.1016/j.visres.2010.07.008 20638401 10.1016/j.visres.2010.07.008 PMC2948855

